# Improving intravenous drugs administration in ICU and reducing drug incompatibilities: a nurselead interdisciplinary program

**DOI:** 10.1186/2197-425X-3-S1-A720

**Published:** 2015-10-01

**Authors:** M Lagabrielle, C Rocchietti, M Sevin, S Calvino-Gunther, A Behouche, C Chapuis, I Messant, M Veteau, C Schwebel

**Affiliations:** CHU Grenoble, Reanimation Medicale, Grenoble, France; CHU Grenoble, Pharmacie, Grenoble, France; Université Grenoble-Alpes, Inserm U1039, Laboratoire des Radiopharmaceutiques Biocliniques, Grenoble, France

## Introduction

Many intravenous (IV) drugs are required for complex and polypathologic critically ill ICU patients management. ICU nurses are daily confronted with drugs preparation and administration (solvent type and volume, infusion rate). They also have to deal with drugs physiochemical incompatibilities (PCI), especially as various IV medications are delivered via a single catheter lumen. PCI may induce a loss of drug efficiency, catheter thrombosis and embolism. Data about drugs PCI in ICU are scarce (1).

## Objectives

To assess nurses' knowledge about IV drugs administration and to evaluate drug incompatibilities before and after a multifaceted intervention.

## Methods

Prospective monocentric interventional study conducted in a 18 bed ICU, divided in 2 phases.

Phase 1- survey of nursing practices by using:

a) semi-directive interviews targeting the whole nursing staff according to general daily practice,

b) direct observation of all patients lines and manifolds by two independent observers twice a week for 3 weeks, in order to unveil drug incompatibilities.

Multifaceted intervention resulted in: computerized prescription order entry (CPOE) update by nurses, pharmacists, and physicians; online course for nurses and physicians; posters; feedback and information to the entire ICU staff.

Phase 2: Post intervention evaluation using the same tools as phase 1 (semi directive interviews and direct observations).

## Results

48 nurses (98% of nurse's team) reported their practices in phase 1 and 2. 56 patients (121 observations, 1072 medications) vs 39 patients (120 observations, 1174 medications) were observed during phase 1 and phase 2 respectively. 89% of the staff (nurses and physicians) attended the online course. Medications involved in PCI showed different pattern before (antibiotics, nicardipine, unfractioned heparin) and after (sedatives /curares, parenteral nutrition, unfractioned heparin) intervention. In phase 1, patients had 2,7 IV lumen on average, and 3,2 in phase 2. PCI still occurred despite preventive measures (at least one drug PCI in 20% of the observation in phase 1 vs 22% in phase 2). Typology of patients, high nurses turnover, restricted number for IV access may have been limiting factors. Nevertheless, standardization of drugs preparation and educational tool improved nurses' knowledge and developed a real team awareness about drugs PCI.

## Conclusion

Drug incompatibilities are common in ICU and mainly occur with drugs used daily. ICU patient's typology and the high number of IV medications are limiting for strict application of pharmaceutical recommendations. Managing incompatibilities of IV drugs for these patients is challenging. Further study aiming to test the impact of 4 lumen catheters, (in association with a multifaceted program), in reducing drug PCI is on going.
